# Bibliographical Notices

**Published:** 1838-04-11

**Authors:** 


### BIBLIOGRAPHICAL NOTICES.

Lectures on Lithotomy, *Delivered at the New York Hospital, December*, 1837. By Alexander H. Stevens, M. D. Surgeon to the New York Hospital, and Emeritus Professor of Clinical Surgery. *New York: Adlard & Saunders*, 1838, pp. 93. [*With five Lithographs.*]

In 1836 a *grand folio*, splendidly illustrated with ten plates, and produced with much typographical magnificence, appeared in Paris with the *affiche* of a "*Memoire sur une maniere nouvelle de pratiquer l'Operation de la Pierre.*" This was a posthumous production of the late Baron Dupuytren, brought to light under the auspices of his literary executors, M. M. Sanson and Begin, by whom indeed it was completed, its illustrious author dying before it was finished. The *Nouvelle Methode* proved however, to be a resuscitation of the most ancient kind of lithotomy, which was practised more than two thousand years ago by Ammonius and Meges, at Alexandria and Rome, in the Augustan Age, and of which so accurate a description has been given us by Celsus, (§ *lib.* 7. *cap.* xxvii.) that it has been named *Lithotomia Celsiani.* The operation is mentioned more or less minutely in all the old surgical works. At the close of the seventeenth century, Rau of Amsterdam, probably performed the "apparatus minor," (as this method was styled by Marianus Sanctus, in contradistinction to that of his master, Johannes de Romanis, which required a chest of instruments) with the improvement of a grooved staff, which was perhaps suggested to him by Frère Jaques, who was at that time operating in Paris. Rau himself was remarkably taciturn respecting his method, and refused to answer any inquiry. Many have given the credit of the addition of a staff, in the Celsian operation, to Fabricius Hildanus. Raoux performed it in France about one hundred and fifty years ago. Heister described and successfully practised it, and it is said that it was, in some instances, adopted by Frère Jaques himself. M. Dupuytren lays no claim to its paternity, having only revived an old and almost forgotten operation, at the same time modifying and adapting it to the present advanced state of anatomical and surgical science.

Conceiving that everything which offers a reasonable prospect of lessening the amount of human suffering as worthy of being received with respect and examined with attention, we shall endeavour to present our readers with an accurate and intelligible account of the *Bi-lateral Operation*, which forms the subject-matter of the work which heads this article. We shall first describe the operation as detailed by Dupuytren; next we shall state the advantages claimed for it by its advocates; examine into the validity of its asserted superiority; compare its results with the average success obtained by the usual means; and, in conclusion, we shall speak of the several instruments employed in its performance.

The three steps of the operation are, first, to expose the urethra by an incision through the integuments, fascia and muscles; secondly, to open the canal; and thirdly, to divide the neck of the bladder and prostate. To accomplish this, the patient is secured in the usual manner, and a curved staff is introduced into the bladder. This is made of wrought steel, with a flat rough ebony handle, having an enlargement in the middle of the instrument, or at the centre of the curve, extending two inches, and is thus accurately adapted to the urethra. The groove is large and deep, with rounded and everted edges, and diminishes gradually until it is lost entirely at the extremity. This is necessary to prevent the arrest of the lithotome, which would occur were its termination abrupt. The staff is now confided to a careful assistant, who maintains it in a vertical direction, the diaphysis of the instrument forming a right angle with the axis of the body, the curved portion being elevated against the symphysis pubis, and not pressed downwards and backwards upon the rectum. The skin, subcutaneous cellular tissue, superficial aponeurosis, and muscular structure are now divided with a double convex-edged scalpel, by a transverse lunated incision across the raphe of the perineum, six lines anterior to the anus, with the horns of the crescent directed towards the os coccygis. Care must be taken that the extent of the wound should correspond throughout its whole depth with the size of the external incision. The knife should pursue the course of an imaginary line extending from the anus to the anterior surface of the bladder and hypogastrium, for, if inclined too much backwards, it must inevitably injure the bulging surface of the rectum. The urethra is entered with the point of the knife, and the groove of the staff exposed for about three or four lines. As at the apex of the urethro-anal triangle the gut is very nearly in apposition with the canal and prostate, great precaution should be observed to keep the point of the scalpel in the groove, to obviate any possibility of its being pierced. The lithotome is now introduced closed, with its convexity towards the sacrum. The contact of the two metallic bodies, communicated to the hand of the surgeon, announces the proper position of the instrument. The surgeon now takes the staff from his assistant, elevates it towards the pubis, and pushes its extremity deeper into the bladder, while at the same moment the lithotome enters the organ along the groove. We are apprised of its presence in the bladder by the gush of urine, and its percussion against the calculus; the staff, on its entry being ascertained, is withdrawn. The lithotome is now turned completely round in the bladder, so that its concavity is towards the rectum. The probable dimensions of the stone are determined, and the screw limiting the extent of divergence of the blades, is adjusted. The centre spring is pressed firmly upon by the surgeon's finger, and the instrument with the expanded blades, is slowly withdrawn, cutting out its own way, the handle being depressed. The operator now puts the index finger of his left hand into the wound, the extent of the incision and condition of the parts are explored, and upon it, as a guide, the forceps are introduced.

We shall now briefly enumerate the advantages claimed for the bi-lateral operation, over the common method, by its partizans. They say that it is simpler, easier executed, and equally safe; that the incision being made at the greatest diameter of the inferior strait, gives an opening of more width; and that it offers the most direct route to the bladder. From these circumstances, they contend, the calculus is more readily discovered, seized and extracted; that, when it is friable and breaks, the shortness and capacity of the passage enable the fragments to be washed out with greater facility and certainty. That the opening being proportionate to the volume of the calculus, there is less risk in withdrawing it of lacerating the surrounding soft parts, and, consequently, the chances of subsequent inflammation diminished. That the limits of the prostate are never exceeded; that the ejaculatory ducts are guarded from any possibility of injury, as well as the large vessels; that there is less danger of wounding the rectum, from the direction of the cut being nearly parallel to that viscus; and finally, that it is adapted to all ages and both sexes.

Let us now determine the value of these statements. Of the fatal cases of lithotomy, two-thirds, it is estimated, die from the effect of inflammation in organs more or less remote from the implicated viscus. Nearly one-fourth perish from hemorrhagy, and the remainder from constitutional irritation, and other as incomprehensible and latent causes. Of those who are destroyed by inflammatory disorders, the seat of these, in the majority of cases, is found to be in the immediate neighborhood of the wound. Examinations after death detect the pelvic viscera, the adjacent serous membrane, and the perineal structure in a state of phlogosis and disorganization. We find the neck of the bladder and the prostate in one of two conditions: either imperfectly divided and severely contused, or, the incision prolonged through the gland into the sub-peritoneal cellular tissue, giving rise to urinary infiltration with its inevitably fatal consequences. Would not these facts then countenance the idea, that the spot usually selected for the performance of the lateral operation, is too narrow, and does not permit an incision of adequate latitude for the extraction of the calculus, without dangerous violence? So long as an instrument so abominably murderous and heathenish as the *gorget* defiles the case of the lithotomist, these evils may be anticipated to a greater or less extent, according as good or bad fortune prevails. While, if the long narrow-bladed knife, now almost universally used in Great Britain, were employed, the incision may be prolonged outwards and backwards to any requisite extent, without risk of trenching on the adjoining cellular texture. To the bi-lateral method must be conceded the superiority, over that with the gorget; whereas if the knife be employed, we conceive that no especial claim can be set up in its favour above the usual lateral section. Its incontestible advantages are, the much less risk of hemmorhagy, there being no danger of wounding the large vessels of the perineum, as well as the incomplete division of the prostate not involving the venous plexus around the gland; and the entire freedom from any chance of emasculation, the seminal ducts not being implicated.

In comparing the actual mortality between the two methods, from separate tables given by M. Dupuytren, we find the balance rather against the bi-lateral operation. Of 356 operations according to the old plan, 61 died, or about 1 in 6; whereas, out of 89 cases in which the *Nouvelle Methode* was practised, 19 died, or 1 in 4 2-3. But M. Velpeau, in a discussion some time since before the French Academy of Medicine, stated that M. Dupuytren lost only 1 in 12, by his bi-lateral section. This compares very favourably with the average mortality from the lateral operation in Paris, which vastly exceeds that of Great Britain or the United States. Sir Astley Cooper estimates that 1 patient in every

8, usually perishes after the operation of lithotomy. Cheselden lost out of 213, 20; or 1 in 10 5-8; Of the first hundred, 6 only, died; of the third fifty, 8; and of the remaining sixty-three, 6. During a period of sixty years, 704 patients were cut at the Norwich Hospital; of these 1 in 8 died. At the Leeds Infirmary, from 1767 to 1817, the mortality was 1 in 7 4-5, according to Dr. Prout; and at the Bristol Infirmary, 1 in 4 1-4. Chelius, in Germany, lost 1 in 22; Petrunti, at Naples, 1 in 25, in private practice; Martineau, 2 in 84, do.; Santuro, 1 out of 56; Vinicel 3 in 83; Pansa, 1 in 70; Ouvrard, 5 in 60. Pajola, of Venice, a pupil of Le Cat, states that he cut 550 patients for stone, with entire success.

In this country the results of several individual operators have been very happy. Of 153 cases cut by Dr. Dudley, 4 only died, or 1 in 38 1-4; Dr. Smith lost 1 in 18 operations. It is much to be regretted that no record has been left by the late Dr. Physick of the number or result of his cases.

We have no intention of agitating here the vexed question of the respective merits of Lithotomy and Lithotripsy, yet we confess ourselves one of those who cherish, and we think with a reasonable prospect of ultimate realization, the hope of ere long seeing the former operation attempted only on extraordinary occasions, when some eccentric annuitant, weary of life, wishes to give himself a chance of ending his "sea of troubles."

It now remains for us to describe the instruments proposed for the section of the neck of the bladder and prostate in the bi-lateral operation. These are the double *lithotome* of M. Dupuytren, and the *Prostatic Bisector* of Dr. Stevens. The incision could be effected perfectly by either the common probe-pointed bistoury, or the *lithotome caché* of Frère Côme, but a double introduction of the instruments would become necessary, delaying needlessly the operation. The instrument of M. Dupuytren is a modification of the common *lithotome*, by M. Charrière, and is simply this. There are two long narrow curved blades, with their cutting edges looking outwards; these fold on each other in a slightly curved case. A spring is attached to the handle, which being pressed upon, the blades quit their sheath and expand to the extent required, which is regulated by a screw, to which an index is attached.

Dr. Stevens' instrument is described by him as follows:

"I venture to offer to the profession a new instrument for the bilateral section of the prostate; in form it resembles a large olive, with a beak at the extremity, with cutting edges at the sides, parallel to its longest axis, and with a straight handle. The instrument, of which there are three sizes, and the manner of employing it, will be readily understood by the annexed engravings. The grooved staff employed in connexion with this instrument, is as wide as the urethra will admit, and the groove gradually terminates as it approaches the end of the staff.

The advantages in the use of this instrument are, first, that the circular form of a transverse section, gives an opening through the gland of three diameters instead of two, as when a flat instrument is employed;—thus it is not necessary to carry the incision so far laterally to obtain an opening of given dimensions; and hence there is less likelihood of hemorrhage from injuring the plexus of vessels that surrounds the prostate.

"Second. The prostate is cut horizontally, and though not absolutely, yet for all practical purposes in its greatest diameter.

"Third. The rectum is pushed back by the convexity of the posterior part of the instrument.

"Fourth. As the prostate is stretched transversely across the instrument, the section is made by a clean cut, and with so little resistance that the instrument does not, like ordinary gorgets, require to be thrust in with force, but may be passed lightly along until the section is completed. Thus there is less danger of wounding the fundus of the bladder by a sudden cessation of resistance from the parts divided; they are, in fact, divided without force.

"Fifth. The easy division of the prostate obviates the danger of tearing the cellular tissue which connects the anterior surface of the bladder to the posterior wall of the ossa pubis." pp. 53-4.

Dr. Stevens thinks "that the double lithotome of Dupuytren ought not to be employed by any one who is not thoroughly drilled in the use of it. He must handle it as he would a double barreled hair-trigger pistol, and be prepared to lacerate the urethra, while passing its beak along the groove of the staff;" and in support of this assertion, cites Sir Astley Cooper on the authority of Dr. Dudley. From not knowing the circumstances of the case, we cannot of course give any positive opinion, but are confident that the Nestor of British surgeons never used the double lithotome of Charriere, and that if there is any foundation for the story, the instrument alluded to must have been the lithotome cachè of Frère Côme. Still we are unwilling to believe that any one of Sir Astley Cooper's acknowledged manual dexterity could have boggled so shamefully as is here represented, and suspect that there is some mistake in the matter. Dr. Stevens is singularly unfortunate in his comparison of the instrument to a "double barreled hair-trigger pistol," inasmuch, as at all times, the blades are completely under the control of the surgeon, and collapse instantaneously when the pressure of the finger is removed. As to the risk of lacerating the urethra, if the proportion between the size of the lithotome and the groove of the staff, be maintained, this is nearly impracticable, except by a very rough manipulator. Dr. S. thinks, that the opening of a "cutting instrument, even with probe points in a contracted bladder, to be attended with risk." This objection, we conceive, applies with ten-fold force against his invention. He considers the "attempt to make the incisions spheroidal as a useless refinement." The incisions made by M. Dupuytren's lithotome are *inevitably* spheroidal, and constitute the distinguishing feature and superiority of the operation, the free division of the gland facilitating the extraction of the calculus. Dr. Stevens' bisector defeats the main object of the *Nouvelle Methode*, and had he employed either the double-lithotome, or even the *bistouri boutonné*, he would probably not have encountered the difficulty of extracting the calculus which he mentions in his first case. The division of the prostate is not effected at even its greatest diameter, and consequently the incision is not as free as the one obtained by the knife of Cheselden, or that of Sir William Blizard, in the simple lateral section. From the wedge-like shape of the instrument, notwithstanding its cutting edges, the wound inflicted in its passage through a dense resisting medium like the prostate, must partake, to a greater or less degree, of the nature of a penetrating or punctured wound, and the tissue of the gland will be more or less compressed, and even contused.

Dr. Stevens has made some other trifling modifications upon M. Dupuytren's *Nouvelle Methode*, which we cannot conscientiously pronounce improvements, or even recommend their trial.

Dr. Stevens' work deserves certainly a careful perusal, and he himself is entitled to the credit, so far as we have ascertained, of having introduced the *bi-lateral operation* to the notice of the profession in this country. He has as yet employed it himself in two cases only, both of whom were children; the result was successful. We think that the operation possesses strong claims on the attention of the American surgeon, as one which in many instances he may find desirable to practise.

Dr. Stevens announces several other lectures in preparation. These we hope speedily to see presented to the public, trusting at the same time that they will be free from the innumerable typographical errors which disfigure the volume before us.

---

*An Experimental Investigation into the Functions of the Eighth Pair of Nerves, or the Glosso-Pharyngeal, Pneumegastric, and Spinal Accessory.* By John Reid, M. D., Fellow of the Royal College of Physicians of Edinburgh, Lecturer on the Institutes of Medicine, formerly Demonstrator of Anatomy, &c. [From the Edinburgh Medical and Surgical Journal. January, 1838.]

This is no essay of an incipient vivisector, but a production of no ordinary character, and meriting from the student of physiology an attentive examination. Dr. Reid's aptitude for the delicate task he has undertaken is of the highest order. His extensive and accurate anatomical knowledge, combined with great manual dexterity, peculiarly fitted him for the enterprize. This paper is characterized by candid statements, cautious deductions, and discriminating reasoning. The exact and ingenuous manner in which all his inquiries are conducted, together with the extraordinary parity of the results obtained, are calculated to inspire the reader with confidence. Independent of a careful repetition of all the experiments, the condition of the parts implicated, were invariably explored after death, to preclude any possibility of deception. He reconciles the discrepancies of previous experimentalists with much acuteness and plausibility. Our author states that "In entering upon this investigation he had no favourite theory to defend, stood committed to no pre-conceived notions, or shackled by any slavish deference to authorities, but was ready and willing to give up any of his former opinions as soon as they appeared inconsistent with the phenomena which presented themselves."

We shall now offer an abstract of this valuable communication to the reader. Our limits will not permit us to enter into the experimental details with any minuteness, and we shall in general avail ourselves of Dr. Reid's resumè.

Our author adopts the old classification of the nervous system, and under the Eighth Pair includes the Glosso-Pharyngeal, Pneumogastric, and Spinal-Accessory.

Glosso-Pharyngeal Nerve. Twenty seven experiments were performed on dogs, to determine the functions of this nerve. "Seventeen of these were for the purpose of ascertaining if it were to be considered a nerve both of *sensation and motion;* and what are the effects of its section upon the *associated movement of deglutition*, and on the *sense of taste*." In the remaining ten, the animals were immediately previous deprived of sensation, in order that its property as a *motor nerve* might be satisfactorily settled.

Previous to announcing the results, we shall premise the manner adopted in conducting the experiments, in order that the reader may assure himself of the extreme care observed in all the investigations, and that they may serve as guides to those who may hereafter feel inclined to pursue the same field of inquiry.

"An incision varying in length, in proportion to the size and species of the dog, was made nearly parallel to the back part of the base of the *inferior maxilla*; its posterior termination always extending a short distance over the anterior margin of the *sterno-mastoid muscle.* After cutting through the skin and *platysma myoides*, or *cutaneous* muscle, the two large veins which go to form the *external jugular* were each secured in a double ligature, and cut across between the points tied. The *glands* at the angle *of the jaw* were removed or dissected aside, and the *carotid artery* exposed. The artery was generally first secured in a ligature below the *hypoglossal nerve*, and before it had given off any of its large branches; it was then exposed above the *hypoglossal*, included in a double ligature and cut across. This was found to be the most effectual method of preventing the troublesome hemorrhage, which otherwise is apt to occur from wounding the large arteries, arising from the carotid at this point. After this, by a little dissection, the nerve was exposed as it lies upon the lower margin of the *stylo-pharyngeus muscle*, near its origin from the styloid process. One of the best guides in displaying the nerve, is the *osseous expansion*, connected with the *tympanic cavity* of the *temporal bone*, which is always readily felt after the *carotid* has been exposed. In dissecting the nerve back to the *foramen lacerum posterius*, great care must be taken to separate it from the *pharyngeal branch of the par vagum*; which lies sometimes in immediate contact with it, at other times, one or two lines below, and is frequently united to it by a considerable communicating branch, so that it may readily be mistaken for a large pharyngeal branch of the glosso-pharyngeal. I am afraid, for reasons to be afterwards stated, that sufficient precautions have not been taken by other experimenters to separate these from each other. This is the more necessary, as I am confident that these two nerves differ very materially in function, and this must consequently have seriously affected the results. The superior laryngeal branch of the *par vagum* is placed a short distance below these two nerves, but was rarely seen except when exposed designedly. I have observed very considerable differences in the manner in which the glosso-pharyngeal gives off its branches to the pharynx and fauces; sometimes it begins to give off small filaments soon after it has emerged from the base of the cranium, and continues to give off a number of small branches in succession, until it has furnished all the filaments to the pharynx and fauces; more commonly the nerve forms a sudden enlargement upon the lower margin of the osseous expansion of the tympanic cavity, from which the principal branches to the pharynx and fauces are given off, one or two of which are much larger than the others. It is almost needless to add, that in all the animals kept alive, after section of these nerves for subsequent observations, a considerable portion of the trunk of each nerve was removed." p. 111-2.

Is the *glosso-pharyngeal*, a nerve both of motion and sensation? On this point Dr. Reid says:

"It was found that the muscular movements excited by pinching this nerve varied in degree in different animals, but were always distinctly marked when the precaution was taken to irritate the nerve before it had given off its pharyngeal branches. It was also repeatedly observed, that when the nerve was cut across, irritation of the lower end, or that in connection with the muscles, was followed by no movement; while, on the other hand, irritation of the cranial end, even when the nerve was divided before it had given off a single twig, was followed by as powerful convulsive twitches, as when the nerve was entire. We also remarked more than once, that while the pinching of the lingual branch caused no muscular movements, irritation of one of the pharyngeal twigs was followed by very strong convulsive twitches of the throat and lower part of the face. The capability of this nerve to transmit those impressions which excite *sensation*, was amply proved by the severe and undoubted indications of suffering which in many cases followed the pinching of it with the forceps. In several cases, however, the indications of suffering were much less strongly marked than in others, but that this nerve possesses *common sensation*, in the usual acceptation of that term, I am fully convinced, not only from the results of these seventeen experiments expressly made upon these nerves, but from five others, where they were exposed in displaying the pharyngeal branches of the *par vagum*."—p. 116-7.

These conclusions are in direct variance with the statements of Panizza, Hall, and Broughton, while they support those of Dr. Alcock. Dr. Reid thinks that these gentlemen all operated on different nerves, and thus reconciles the discrepancies of their several views. This he thinks explains the fact, "without seriously impugning the anatomical knowledge, or the acuteness of observation of either party; for," continues he:

"I have no hesitation in saying, that if in these three experiments (Exps. v. vi. and vii.) and in others not detailed here, we had operated on that part of the trunk of the nerve which first presented itself, and not proceeded to dissect it back towards its place of exit from the cranium, we should have gone away with the impression that the irritation of this nerve was followed by no muscular movement, and little if any indications of suffering." p. 117.

Do the muscular movements of the throat and inferior part of the face, which are consequent upon the application of a mechanical or chemical irritant to the glosso-pharyngeal nerve depend upon an inherent motive power, or do they arise from a transmission of the impression along the filaments of sensation to the nervous centres, which being received there, is reflected on the contractile tissue through adjacent motor nerves? Upon this question Dr. Alcock is undecided, and hazards no positive opinion. Our author appears satisfactorily to have settled this mooted point, he says:

"In the experiments which I have detailed above, we have, however, sufficient evidence to decide that these muscular movements can only be explained on the supposition that they depend upon some influence transmitted along other nerves distributed to these muscles, and through the intervention of the central organs of the nervous system. We there find, and the facts were still further confirmed by what we observed in other experiments, that irritation of a single pharyngeal twig, or, what is still more conclusive, of the upper or cranial end of the cut nerve, before it had yet given off any of its branches, was attended by as powerful convulsive movements, as when the trunk of the nerve and all its branches were entire and uninjured." p. 119.

Both Mr. Mayo and Müller have contended that the glosso-phryngeal possesses duplicate properties, and that it is both a motor and sensitive nerve, and each has maintained his assertion on separate grounds. Dr. Reid repeated Mr. Mayo's experiment on ten dogs. The nerve was exposed and irritated immediately after death, and though the results were not uniform, there was no satisfactory evidence elicited to sanction the belief that the glosso-pharyngeal was a nerve of motion; no muscular movements were seen on irritating this nerve. The irritation of the pharyngeal branch of the *par vagum* was the test that muscular contractility remained in sufficient vigor to be excited readily through the motor nerves, and also as a standard of comparison. Mr. Mayo's error, it appears, probably arose from the nerve experimented on by him being the pharyngeal branch of the *par vagum*, which lies close to the *glosso-pharyngeal*, the trunks of the two nerves anastomosing frequently by a short branch anterior to the *foramen lacerum*; in which case the pharyngeal branch of the *par vagum*, may, very readily, be confounded with the glosso-pharyngeal nerve. In one subject, which Dr. Reid dissected, a communicating branch connected the trunk of the nerves, before they had fairly cleared the *foramen lacerum*. This intimate association was most likely the source of fallacy in Mr. Mayo's experiment.

Professor Müller's argument, in favour of the compound property, of this nerve is founded on anatomy, and he reasons from its analogical structure to the spinal nerves. The existence of the *ganglion jugulare* of Ehrenritter is beyond doubt, and is probably limited to the posterior root, but the *ganglion petrosam* of Andersh, involving the whole trunk, and situated immediately below the former, impairs the exactness of the resemblance.

The next subject of inquiry is, what effect the section of the glosso-pharyngeal nerve has upon the *functions of deglutition.*

Sir Charles Bell regards the function of this nerve as associating the movements of the tongue and pharynx with the muscles of respiration in the instinctive movements of deglutition. Dr. Alcock, of Dublin, confirmed these views. Dr. Reid invalidates the statements of both of these gentlemen, and proves pretty conclusively that the latter divided the pharyngeal branch of the *par vagum* along with the glosso-pharyngeal, the relation of these two nerves, as before remarked, being so intimate, that they can be only safely distinguished by tracing them to their origin. Dr. Reid experienced much difficulty in effecting the division of the nerve before any twigs had been given off, and it was only after repeated failures that he at last succeeded. Careful *post-mortem* inspection of the parts was made in every instance. So soon as the inflammation and swelling consequent on the injury had subsided, before any possibility of reunion could occur, the animal was enabled to swallow with integrity.

The last subject of investigation is, how the division of this nerve affects the sense of taste. Panizza, Marshall Hall, and the late Mr. Broughton have all endeavored to establish this as the special nerve of taste; while Alcock, and Kornfield of Berlin, have denied to it this function exclusively. Dr. Reid's experiments confirm in every particular the observations of the Dublin physiologist. A portion of the trunk of the nerve was removed on both sides in several dogs, and in all instances a persistence of the sense of taste resulted. Coloquintida was rejected, if offered by itself, but if the animal was very hungry, and the bitter was sprinkled on meat, rather than loose the latter he swallowed both, if he thought he had no chance of obtaining the unadulterated article. Where the nerve was cut on one side only, he ate readily bread dipt in a strong infusion of gentian root. In one case, where the nerve beyond all question was completely divided before a single filament was given off, the dog swallowed eagerly animal food, but a morsel, exact in size and appearance, in which the coloquintida was so concealed as to be detected by taste alone, was instantly rejected. Dr. Reid however, never unreasonably exclusive in his doctrines, thinks it probable that the glosso-pharyngeal nerve may participate in the function of taste, in connection with the lingual division of the third branch of the fifth, and the palatine twigs of the second of the fifth. Dr. Alcock, in his sixth report to the British S. Association, having proved this point pretty conclusively, and it being also supported from the anatomical fact of the mucous membrane and papillæ of the tongue, for one inch anterior to the foramen cœcum, being supplied nearly exclusively by this nerve.

From a review of all the experiments performed on the *glosso-phyrangeal nerve*, by Dr. Reid, he is led to infer the subjoined conclusions.

1. "That this is a nerve of *common sensation*, as indicated by the unequivocal expression of pain by the animal when the nerve is pricked, pinched, or cut.

2. "That mechanical or chemical irritation of this nerve, before it has given off its pharyngeal branches, or of any of those branches individually, is followed by extensive *muscular movements* of the throat and lower part of the face.

3. "That the muscular movements thus excited, depend, not upon any influence extending downwards along the branches of the nerve to the muscles moved, but upon a *reflex action*, transmitted through the central organs of the nervous system.

4. "*That these pharyngeal branches of the glosso-pharyngeal* possess endowments connected with the *peculiar sensations* of the mucous membranes upon which they are distributed, though we cannot pretend to say positively in what these consist.

5. "That this cannot be the sole nerve upon which all these sensations depend, since the perfect division of the trunk of the nerve on both sides does not interfere with the perfect performance of the *function of deglutition.*

6. "That mechanical or chemical irritation of the nerve, immediately after the animal has been killed, is not followed by *any muscular movements*, when sufficient care has been taken to insulate it from the *pharyngeal branch of the par vagum.* And we here again observe an important difference between the movements excited by irritation of the *glosso-pharyngeal* and those of a *motor nerve.* For while the movements produced by the irritation of the *glosso-pharyngeal* are arrested as soon as the functions of the central organs of the nervous system have ceased; those, from irritation of a motor nerve, such as the *pharyngeal branch of the par vagum*, continue for some time after this, and when all connection between it and the *medulla oblongata* has been cut off.

7. "That after perfect section of the nerve on both sides, the *sense of taste* is sufficiently acute to enable the animal readily to recognize bitter substances.

8. "That it probably may participate with other nerves in the performance of the function of taste, but it certainly is not the special nerve of that sense.

"Lastly, the *sensation of thirst*, which is referred to the *fauces* and *pharynx*, does not appear to depend entirely on the presence of this nerve. The animals in which it was divided lapped water of their own accord. I observed one of them in which the nerves were found satisfactorily divided, rise, though very feeble, walk up to a dish containing water, lap some of it, and return again to the straw on which he was previously lying." p. 129-30.

(*To be continued.*)

